# Nucleus Accumbens Deep Brain Stimulation Results in Insula and Prefrontal Activation: A Large Animal fMRI Study

**DOI:** 10.1371/journal.pone.0056640

**Published:** 2013-02-18

**Authors:** Emily J. Knight, Hoon-Ki Min, Sun-Chul Hwang, Michael P. Marsh, Seungleal Paek, Inyong Kim, Joel P. Felmlee, Osama A. Abulseoud, Kevin E. Bennet, Mark A. Frye, Kendall H. Lee

**Affiliations:** 1 Department of Neurologic Surgery, Mayo Clinic, Rochester, Minnesota, United States of America; 2 Department of Neurosurgery, Soonchunhyang University, Busheon Hospital, Bucheon, Republic of Korea; 3 Department of Radiology, Mayo Clinic, Rochester, Minnesota, United States of America; 4 Department of Psychiatry and Psychology, Mayo Clinic, Rochester, Minnesota, United States of America; 5 Division of Engineering, Mayo Clinic, Rochester, Minnesota, United States of America; 6 Department of Physiology and Biomedical Engineering, Mayo Clinic, Rochester, Minnesota, United States of America; University of Minnesota, United States of America

## Abstract

**Background:**

Deep Brain Stimulation (DBS) of the nucleus accumbens (NAc) has previously been investigated clinically for the treatment of several psychiatric conditions, including obsessive-compulsive disorder and treatment resistant depression. However, the mechanism underlying the therapeutic benefit of DBS, including the brain areas that are activated, remains largely unknown. Here, we utilized 3.0 T functional Magnetic Resonance Imaging (fMRI) changes in Blood Oxygenation Level-Dependent (BOLD) signal to test the hypothesis that NAc/internal capsule DBS results in global neural network activation in a large animal (porcine) model

**Methods:**

Animals (n = 10) were implanted in the NAc/internal capsule with DBS electrodes and received stimulation (1, 3, and 5 V, 130 Hz, and pulse widths of 100 and 500 µsec). BOLD signal changes were evaluated using a gradient echo-echo planar imaging (GRE-EPI) sequence in 3.0 T MRI. We used a normalized functional activation map for group analysis and applied general linear modeling across subjects (FDR<0.001). The anatomical location of the implanted DBS lead was confirmed with a CT scan

**Results:**

We observed stimulation-evoked activation in the ipsilateral prefrontal cortex, insula, cingulate and bilateral parahippocampal region along with decrease in BOLD signal in the ipsilateral dorsal region of the thalamus. Furthermore, as the stimulation voltage increased from 3 V to 5 V, the region of BOLD signal modulation increased in insula, thalamus, and parahippocampal cortex and decreased in the cingulate and prefrontal cortex. We also demonstrated that right and left NAc/internal capsule stimulation modulates identical areas ipsilateral to the side of the stimulation

**Conclusions:**

Our results suggest that NAc/internal capsule DBS results in modulation of psychiatrically important brain areas notably the prefrontal cortex, cingulate, and insular cortex, which may underlie the therapeutic effect of NAc DBS in psychiatric disorders. Finally, our fMRI setup in the large animal may be a useful platform for translational studies investigating the global neuromodulatory effects of DBS

## Introduction

The discovery of chlopromazine in 1952 by the French Surgeon and anesthesiologist Henri Laborit [1] sparked the new era of psychopharmacology and set the stage for biological treatment of various psychiatric illnesses. However, after half a century, the efficacy of our current therapeutic agents remains suboptimal and patient’s adherence to treatment is often hampered by considerable side effect profiles. As a result, deep brain stimulation (DBS) of the nucleus accumbens (NAc) is emerging as an effective treatment for reducing symptom severity in obsessive compulsive disorder (OCD) [2], [3], [4], [5], Tourette’s syndrome [6], [7], [8], [9], major depressive disorder [10], [11], [12], and alcoholism [13]. This practice is also supported by preclinical models, in which NAc stimulation reduces compulsive checking in quinpirole rat models of OCD [14], decreases alcohol consumption in alcohol preferring [15], [16] and attenuates re-instatement in cocaine-seeking [17], and morphine-preference in rats [18].

Despite this well documented preclinical and clinical efficacy, the mechanism of action of NAc DBS remains largely unknown. Recent reports suggest that DBS causes distal axonal network activation [19], [20], [21]. Given the unique anatomical location of the accumbens as the interface between limbic and motor circuitry [22], [23], DBS could facilitate the function of the accumbens in engaging the thalamocortical circuitry essential for translating motivationally relevant information into actual adaptive behavioral responses [23], [24], [25], [26]. Several techniques have been used in attempt to elucidate the effect of NAc DBS on neural activity. For example, electrophysiological recordings have shown that NAc DBS inhibits firing in orbitofrontal neurons in the normal rat model [27]. Likewise, in imaging studies, such as those utilizing 18F-FDG/PET, NAc DBS has been shown to result in decreased metabolism in the subgenual cingulate and in prefrontal regions in patients with treatment resistant depression [28] and OCD [29], [30], [31]. The present study utilizes functional magnetic resonance imaging (fMRI), a technique which provides *in vivo* real-time anatomic maps of blood oxygenation in the brain under normal physiological conditions [32], [33]. fMRI has become an increasingly popular technique to study mechanisms of DBS [34], [35], [36], [37].

Although traditionally the definitive large animal model for translational studies in neuroscience has been the nonhuman primate, the porcine model was selected because the reduced ethical and economic burden enables studies of larger cohorts of animals [38]. Indeed, being similar in size and organization to the brain of the non-human primate [39], [40] the gyrencephalic swine brain, in contrast with the brain of small animals, is more closely representative of the human brain [39], [40], [41], [42]. Specifically, the mean± standard error of the mean (SEM) distance between the anterior commissure and posterior commissure (AC-PC) length for pigs in this study was 12.94±0.30 mm as compared to the 28.3±0.2 mm human AC-PC length that has been reported in the literature. Notably, this porcine AC-PC length is very similar that reported for rhesus monkeys (13.8±0.1 mm) and cynomologous monkeys (12.3±0.1 mm) (Fiandaca et. al. 2011). In particular, the NAc region of the pig is approximately 3.5×5.5×8.5 mm (Felix et.al. 1999) as compared to 14.5×7.0×19.4 mm described for humans (Neto et.al. 2008). Furthermore, several other groups describe the increasing prevalence of pig models in neuroscience [38], [43]. Sauleau and colleagues in particular, highlight the usefulness of the pig as a model of brain imaging techniques, including PET, MRI and fMRI, as well as neurosurgical stereotaxic navigation [43]. Importantly, there is also a growing body of research into the dopaminergic circuitry of pig striatum and the implications of such studies of pig neurotransmission for modeling aspects of human psychiatric disease [38]. Although future studies are needed to further validate this promising large animal model for neuroscientific applications, the ability to perform fMRI in the porcine model enables more precise localization of regions of change with DBS through use of a high-resolution 3-D pig brain atlas [44] to normalize fMRI data from individual pigs for group comparisons [37]. Using this model, the aim of this study was to test the hypothesis that NAc/internal capsule DBS delivered at clinically utilized stimulation parameters in a large animal model affects global fMRI network activation.

## Methods

### Animals

Ten domestic male pigs (30+/−5 kg) were used in this study. They were housed individually in a controlled environment with humidity 45%, temperature 70°F, once daily feeding and access to water ad libitum.

### Ethics Statement

All study procedures were performed in accordance with the National Institutes of Health Guidelines for Animal Research (Guide for the Care and Use of Laboratory Animals) and approved by Mayo Clinic Institutional Animal Care and Use Committee (Protocol A22710).

### Preoperative Imaging

**Figure 1:**
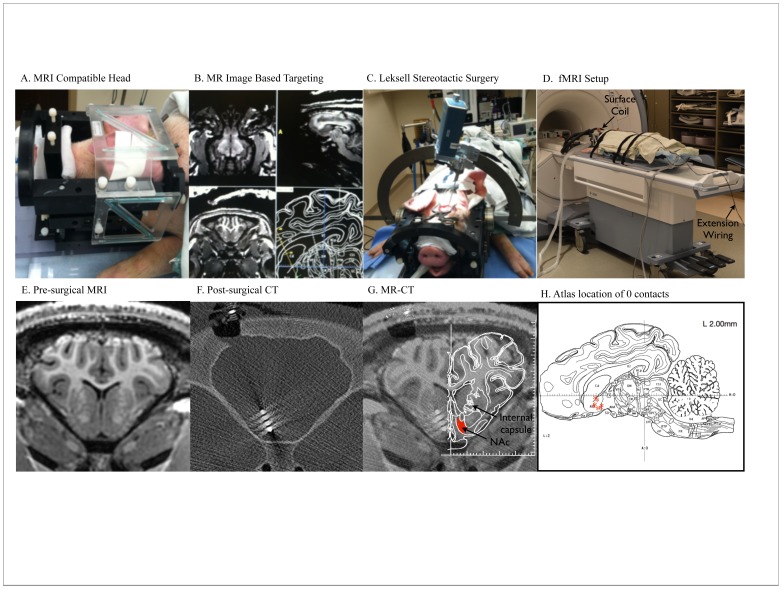
DBS surgery and Lead Confirmation. A) Custom designed MRI-compatible head frame. B) Screenshot of MR-image based targeting procedure using modified COMPASS software. C) Surgical introduction of the Medtronic 3389 DBS electrode using the Lexsell stereotactic arc. D) fMRI Experimental Setup. Extension wiring connected the externalized DBS lead with a pulse generator located outside the scan room. E) Representative pre-surgical anatomical MP-RAGE scan. F) Post-surgical CT scan demonstrating electrode location in the left NAc G) MR-CT fusion with atlas overlay [45]demonstrating the location of the electrode tip in the left NAc. H) Diagram plotting the location of the 0 contact in each animal (red asterisks), as determined by the MR-CT fusion on a stereotaxic pig brain atlas, sagittal slice (lateral 2.00 mm from midline) [45].

A Mayo Clinic-developed MRI-compatible stereotactic head frame, previously described by our group, was used for DBS electrode targeting [40] (Figure 1A). Preoperative anatomical imaging consisted of a 3D MP-RAGE sequence with 1.5 mm slice thickness, 24 cm field of view and matrix of 512×512, performed on a 3.0 T MRI (General Electric Health Care, Signa HDx 16×x software with 23 mT/m gradient set, Waukasha, WI). A custom developed 4-channel phased array receive only coil array was used in this experiment. The coil array was small enough to fit inside the head frame, just above the pig skull and below the localizer box, a set-up that provides excellent signal-to-noise ratio (SNR). DICOM image data were then transferred to a stereotactic planning computer with Compass navigational software, modified to accommodate the pig head frame coordinates (Figure 1B). MRI data was then merged with the pig brain atlas using the anterior-posterior commissural line as a reference [45] and stereotactic coordinates for the DBS electrode implantation trajectory were defined for the left unilateral (n = 9) or bilateral (n = 1) NAc/internal capsule region.

### DBS Electrode Implantation

Animals were administered anesthesia induction with Telazol (5 mg/kg i.m.) and xylazine (2 mg/kg i.m.), followed by intubation and isoflurane (1.5–3%) maintenance throughout the remainder of the procedure. Vital signs (heart rate and temperature) were continuously monitored and respirations were maintained at 12 breaths per minute throughout the procedure. Medtronic 3389 DBS electrode(s) were delivered through a 5–10 mm burr hole using an Alpha-Omega computer-controlled Microdrive attached to a Leksell stereotactic arc secured to the head frame (Figure 1C). The electrode contacts were named from the most distal contact as “0”, “1”, “2” and most proximal as “3”. The electrode was secured to the skull using an anchoring system (Navigus cap, Medtronic). The electrode was positioned so that the NAc was located between contacts 0 and 1. Targeting was landmark-based and located immediately inferior and anteromedial to the anterior commissure. The mean±standard deviation coordinates for contact 0 were: x = 2.53±0.55 mm lateral from the intercommisural (AC-PC) line; y = 5.25±1.23 mm anterior from the AC; z = 4.94±0.68 mm inferior from the AC-PC. The location of the electrode was then confirmed radiographically with post-operative CT (Dual source Somatom Definition, Siemens AG) fused with the preoperative MP-RAGE scan (Figure 1E–G). The resulting MR-CT fusion was overlaid on the stereotaxic atlas from Felix et.al. [45], and the location of the 0 contact was plotted on this atlas (Figure 1H).

### fMRI

**Figure 2:**
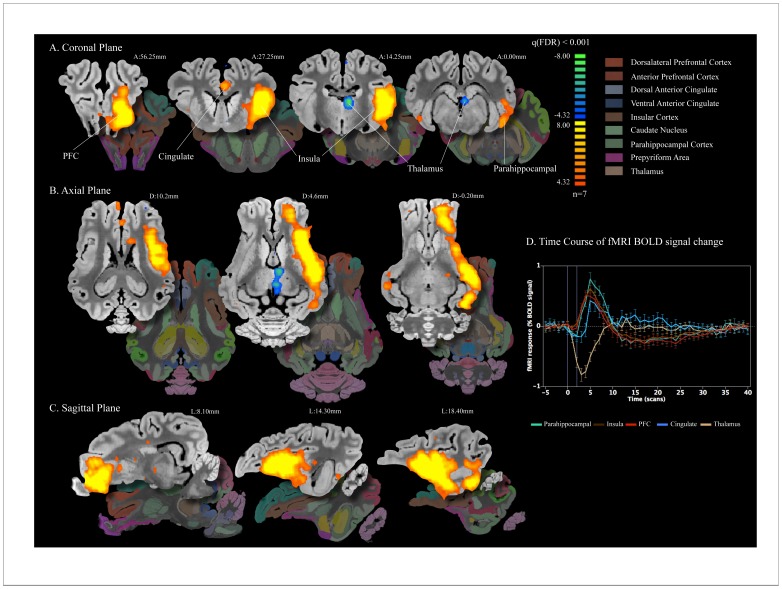
NAc DBS elicits distal network BOLD changes. A–C) Areas of activation with left unilateral NAc stimulation at 5 V 130 Hz 500 µs (n = 7), normalized to a 3D pig brain template [44] Significant activation q(FDR)<0.001 was observed in the anterior and dorsolateral prefrontal (red), insula (brown), parahippocampal (green) and cingulate cortex (blue). Decrease in BOLD signal was observed in the dorsal region of the thalamus (tan). Slice locations are presented in distance (mm) from the posterior commissure. D) Event-related analysis of the average time course for each region of interest was plotted as average percent change in BOLD signal from baseline vs. time (one scan is equal to TR = 3 seconds) using ten frames (30 seconds) prior to stimulation onset as the baseline. Duration of stimulation is marked by the vertical purple lines. In all regions of interest, there is a clear peak in percent change associated with stimulation.

**Figure 3:**
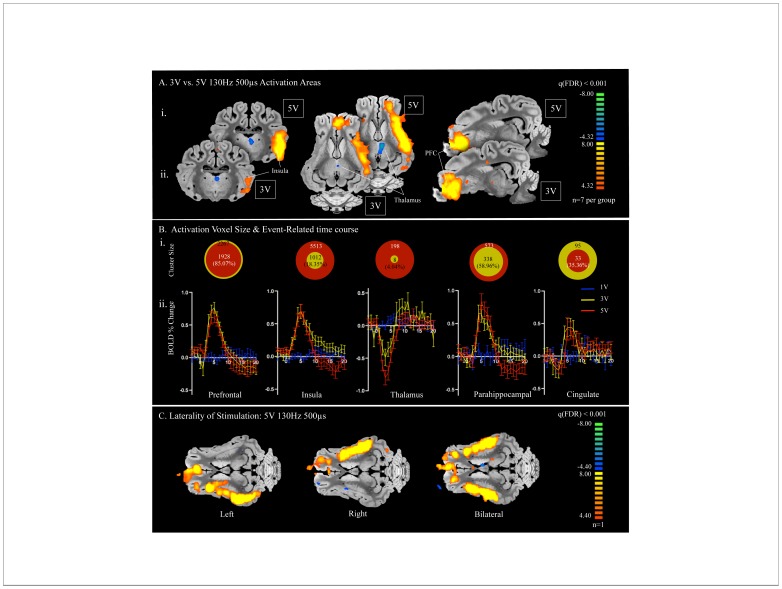
Voltage Dependency of fMRI BOLD signal. A) Comparison of data from left unilateral NAc stimulation at 3 V 130 Hz 500 µs (i; n = 7) with stimulation at 5 V 130 Hz 500 µs (ii; n = 7). Both voltages showed regions of activation in the prefrontal cortex and insula as well as an area of deactivation in the dorsal region of the thalamus. B) i. Region of interest cluster sizes (mm^3^) comparing the percent size of areas of activation with 3 V 130 Hz 500 µs (yellow; n = 7) and 5 V 130 Hz 500 µs (red; n = 7), represented by the relative size of the two circles. ii. Event-related time course of percent change in BOLD signal from baseline with 1 V (blue; n = 5), 3 V (yellow; n = 7), and 5 V at 130 Hz (red; n = 7), 500 µs pulse width. C) Unilateral stimulation to the left (left) right (middle) and bilateral (right) NAc (n = 1). Stimulation of the right NAc activated areas corresponding to those of left NAc stimulation, including prefrontal cortex and insula, ipsilateral to the side of stimulation.

**Figure 4:**
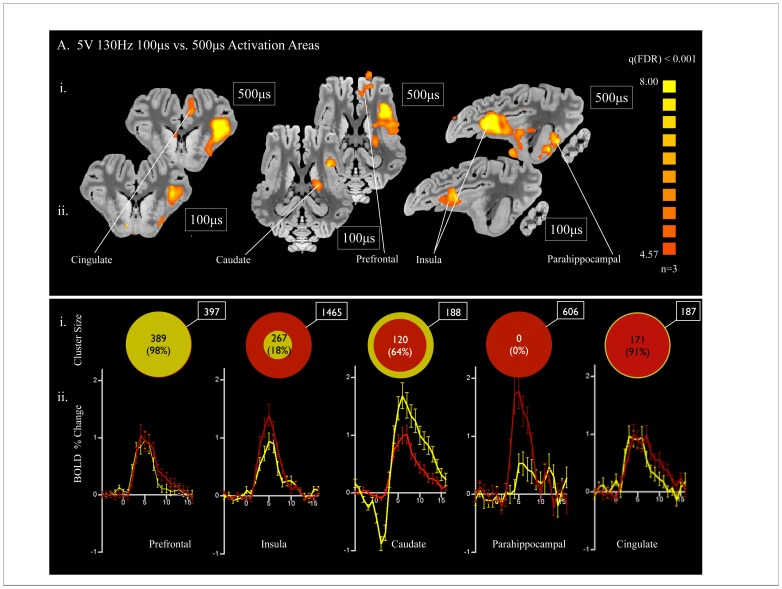
Pulse Width dependency of fMRI BOLD signal. A) Comparison of data from left unilateral NAc stimulation at 5 V 130 Hz 500 µs (i; n = 3) with stimulation at 3 V 130 Hz 100 µs (ii; n = 3). Both pulse widths showed regions of activation in the prefrontal cortex, insula, dorsal anterior cingulate, caudate. There was an additional area of activation in parahippocampal cortex present only with stimulation at 5 V 130 Hz 500 µs. B) i. Region of interest cluster sizes (mm^3^) comparing the percent size of areas of activation with 5 V 130 Hz 100 µs (yellow; n = 3) and 5 V 130 Hz 500 µs (red; n = 3), represented by the relative size of the two circles. ii. Event-related time course of percent change in BOLD signal from baseline with 100 µs (yellow; n = 3) and 500 µs (red; n = 3) pulse widths at 5 V and 130 Hz.

Following implantation of the DBS electrode and CT confirmation, the animal was then returned to the 3.0 T MRI. Extension wiring connected the DBS externalized lead to a programmable pulse generator (A–M Systems Isolated pulse stimulator Model 12100 or Mayo Investigational Neuromodulation Control System (MINCS), an in-house developed wireless stimulation system) located outside the scan room, allowing activation of the DBS electrode during fMRI scanning (Figure 1D). The impedance was checked immediately following the surgery as well as following setup of the animal in the MRI to ensure lead integrity and estimate charge density. This setup was consistent across animals. The animals received stimulation at 1, 3 and 5 V, 130 Hz, and pulse widths of 100 and 500 µsec using a bipolar configuration, applied to the 0(−) and 1(+) contacts of the 3389 lead. During the fMRI scan the heart rate (∼120 bpm) was continuously monitored and no changes were detected with stimulation. To eliminate any movement during the fMRI experiment, the pigs were administered a 2 mg bolus of pancuronium bromide or vecuronium bromide, and maintained with 3 mg/hr throughout the remainder of the experiment. The fMR imaging acquisitions were performed during both DBS “on” and “off” conditions using two-dimensional gradient-echo echo-planar imaging with the following imaging parameters: TR/TE: 3000/34.7, flip angle: 90, FOV: 15 cm×15 cm, matrix: 64×64, slice thickness 2.4 mm with no gap. For each acquisition, 250 volumes were acquired using a block paradigm with five 6 sec periods of stimulation alternated with 120 sec rest periods (Figure 2–3) or 130 volumes were acquired using a block paradigm with five 6 sec periods of stimulation alternated with 60 sec rest periods (Figure 4). Both paradigms were designed to allow the BOLD signal to recover approximately to baseline after each stimulation period.

### Data Analysis and Statistics

Post-processing steps implemented in the Brain Voyager QX software included three-dimensional motion correction and temporal filtering (Gaussian filter; FWHM 3 data points). Data was then normalized to a three-dimensional pig brain atlas [44] and functional activation maps were generated by correlation of the observed signal intensity changes in each voxel with the given stimulus. Double-gamma hemodynamic response functions were used to account for the hemodynamic delay. To correct for multiple comparisons and exclude false positive voxels, we only considered voxels with a significance level less than the False Discovery Rate (FDR) q<0.001 to represent sites of activation. For group comparison, these data-sets were further analyzed with a linear regression analysis (general linear model, multi subject analysis, implemented in Brain Voyager QX software).

## Results

### Validation of Experimental Paradigm

In order to confirm that the experimental setup did not produce artifacts in BOLD signal responses, we assessed a series of control conditions. At subthreshold stimulation parameters (1 V 130 Hz 500 µs), there is no pattern of BOLD signal response to stimulation (Figure 3B). Additionally, we conducted experiments in which we performed DBS electrode implantation into the NAc followed by a sham stimulation fMRI sequence conducted with a nearly identical setup to the experimental conditions, with wires extended from the scan room but not connected to the stimulator outside the scan room during the scanning sequence. There was no BOLD signal response to sham stimulation (data not shown). Together, these results suggest that there was minimal interaction between the RF or gradient pulses and the DBS cabling producing BOLD signal artifacts.

### DBS Surgery and Lead Confirmation

Ten male pigs underwent 3.0 T MR guided placement of a Medtronic 3389 DBS electrode into the NAc through the internal capsule, using the Leksell Stereotactic Arc and Mayo-developed MRI compatible head frame for the pig, as shown in (Figure 1A). Post-surgical CT scans were obtained (Figure 1F). Pre-operative MR and postoperative CT image fusion demonstrated accurate placement of the 0,1 contacts on the DBS electrode into the NAc area, although there is variability in the region of NAc being stimulated between pigs, as seen in Figure 1G–H.

### NAc DBS Elicits Distal Network BOLD Changes

To determine the global neural network activation with NAc/internal capsule DBS, stimulation was first applied to the left NAc/internal capsule, and multi-subject general linear model was performed to determine the areas of increase in fMRI BOLD signal in seven pigs. With application of 5 V, 130 Hz, 500 µs pulse width stimulation to the 0,1 contacts, areas of BOLD signal increase included anterior prefrontal, insula, dorsal anterior cingulate, and parahippocampal cortex (Figure 2A–C). Interestingly, a decrease in BOLD signal in the dorsal region of the thalamus was also observed (region seen in blue in Figure 2A–B).

### Time Course of fMRI BOLD Signal Change

To study the time course of fMRI BOLD signal change in relation to DBS block design, the percent change over five stimulation trials was averaged together within each of the five regions of interest, including parahippocampal cortex, insula, prefrontal cortex, cingulate, and thalamus. As seen in figure 2D, event-related analysis of the average time course for each region of interest was plotted as percent change in BOLD signal vs. time (one scan is equal to TR = 3 seconds) using ten frames prior to stimulation onset as the baseline. The parahippocampal cortex, insula, prefrontal cortex, and cingulate showed stimulation time-locked increase in fMRI BOLD signal, peaking around 5 scans (i.e. 15 seconds) after stimulation onset, whereas the thalamus showed stimulation time-locked decrease in fMRI BOLD signal, peaking around 3 scans (i.e. 9 seconds) after stimulation onset. In all regions of interest, there was a clear peak in percent change associated with stimulation.

### Significant Clusters in the General Linear Model

To quantitate precisely the cluster size and primary peak location with each of the five functionally-defined regions of interest, we performed a general linear model comparing stimulation “on” periods to baseline (Table 1). The largest cluster size (5513 mm^3^) and the most significant voxel (t = 14.03) was detected in the ipsilateral insula, while the smallest cluster size (33 mm^3^) and lowest maximum t-score (t = 6.73) was observed in the ipsilateral cingulate. Consistent with the decrease in BOLD signal seen in Figure 2, the thalamus showed negative maximum t-score (t = −7.77).

**Table 1:** Significant Clusters in the General Linear Model comparing Stimulation “on” periods to baseline.

Locations	Cluster Size (mm×mm×mm)	Primary Peak Location (mm from posterior commissure)	Maximum t-score (within region of interest)	Average p-value (within region of interest)
Prefrontal Cortex (I)	1928	AP 57.25, ML 9.90, DV −6.60	12.57	<0.0001
Insula (I)	5513	AP 20.00, ML 18.60, DV 4.00	14.03	<0.0001
Parahippocampal (I)	573	AP 8.25, ML 18.90, DV −1.50	10.16	<0.0001
Cingulate (I)	33	AP 28.00, ML 2.75, DV 13.80	6.73	<0.0001
Thalamus (I)	198	AP 14.75, ML 2.80, DV 5.80	−7.77	<0.0001

I = Ipsilateral, AP = Anterior/Posterior, ML = Medial/Lateral; DV = Dorsal/Ventral.

Stimulation Parameters = 5 V 130 Hz 500 µs.

### Voltage-dependency of fMRI BOLD Signal

Because in humans there is parameter dependence of DBS effect [46], we varied the stimulation voltage (while holding constant the frequency at 130 Hz and pulse width at 500 µs) to assess the voltage dependency of fMRI BOLD signal in our animal model. As seen in Figure 3, the fMRI BOLD response was measured with stimulations of 1 V (n = 5), 3 V (n = 7), and 5 V (n = 7). In the multi-subject general linear model assessing activation, there was no significant activation at 1 V (data not shown), but there was significant overlap between the regions activated at 3 V as compared to 5 V (Figure 3A). In Figure 3Bi region of interest cluster sizes (mm^3^) comparing the percent size of areas of activation with 3 V (yellow) and 5 V (red) are represented by the relative size of the two circles. Importantly, with increasing voltage, the volume of area modulated increased in the insular cortex, thalamus, and parahippocampal cortex (Figure 3Bi). In contrast, from 3 V to 5 V, the area of prefrontal cortex activation decreased from 2267 to 1928 mm^3^, respectively. Similarly, the area of cingulate activation decreased from 95 to 33 mm^3^, (Figure 3B), reflecting a more prominent deactivation in the prefrontal cortex and cingulate in n = 2 pigs at this higher voltage (data not shown). The event-related time course of percent change in BOLD signal from baseline (Figure 3Bii) showed no pattern of BOLD signal modulation at 1 V (blue). Interestingly, there was a similar pattern of time-locked BOLD signal modulation at 3 V (yellow) and 5 V (red).

### Laterality of Stimulation

Next, to test the idea that the laterality of the stimulation is important [47], we explored the fMRI activation associated with stimulation of the unilateral left, right, or bilateral NAc/internal capsule in one pig. Right-sided unilateral stimulation activated structures symmetric to left-sided stimulation, including the prefrontal cortex and insula on the side ipsilateral to stimulation (Figure 3C). Notably, the area of activation in regions of interest was the same for bilateral stimulation.

### Pulse width Dependency of fMRI BOLD Signal

Finally, to determine the effect of stimulation at more clinically relevant parameters we performed in a separate cohort of three animals a comparison of the 500 µs pulse width such as that applied in the major comparisons of the present study with a lower pulse width (100 µs) within the range those applied in clinical situation [48]. Voltage and frequency were held constant at 5 V and 130 Hz. The regions of activation were similar for both pulse widths and included the prefrontal cortex, insula, dorsal anterior cingulate, and caudate (Figure 4A). However, significant activation was observed in the parahippocampal cortex with the 500 µs pulse width while none was present with the 100 µs pulse width (Figure 4A). The volume of area activated within the insula was significantly larger at 500 µs as compared to 100 µs, with the region activated at 100 µs being only 18% of that activated at 500 µs (Figure 4Bi). The caudate region showed slightly greater area of activation with 100 µs. In contrast, the areas of activation in the prefrontal cortex and cingulate were roughly equivalent at 100 µs and 500 µs (Figure 4Bi). The patterns of BOLD signal percent change were largely similar for both pulse widths in the prefrontal cortex, insula, and cingulate (Figure 4Bii). The caudate, however, showed a different pattern, with a larger initial decrease in BOLD signal followed by a larger increase in BOLD signal percent change, as compared to 500 µs (Figure 4Bii). Finally, in accordance with the lack of activation observed in the parahippocampal cortex at 100 µs, there was no pattern of stimulation time-locked increase in BOLD signal at this pulse width (Figure 4Bii).

## Discussion

In accordance with our hypothesis, we found significant NAc DBS-evoked fMRI BOLD activation in an interconnected neural network, including anterior prefrontal cortex, insular cortex, dorsal anterior cingulate, and parahippocampal cortex. This activation was accompanied by deactivation in the dorsal region of the thalamus. Our results are consistent with studies of internal capsule/ventral striatum DBS using both18F-fluorodeoxyglucose (18F-FDG)/PET and ^15^OH_2_O/PET in humans, which demonstrated activation in the frontal cortex, anterior cingulate cortex, striatum, globus pallidus, and thalamus [28], [49]. Additionally, in a single case study of anterior internal capsule stimulation, fMRI activation was observed in the ipsilateral head of the caudate, medial thalamus, and anterior cingulate and the contralateral cerebellum [28]. Finally, electrophysiological studies in normal animal models have revealed antidromic inhibition of prefrontal cortical neurons and alteration in local field potentials secondary to NAc DBS [27], consistent with our finding of modulation of structures such as prefrontal cortex. Taken together, the results of the current study are consistent with that previously described, in that NAc DBS results in network changes that involve cortical and subcortical areas previously shown to be important in neuropsychiatric disorders. Notably, while activation in subcortical regions near the electrode has been demonstrated in previous studies [28], [49], no such changes were observed in the present study. We hypothesize that this could be due to susceptibility artifact from the DBS leads. Importantly, our study is unique in that it has revealed areas of fMRI BOLD signal change with high frequency stimulation of the NAc/internal capsule across multiple subjects and has presented a novel pig model for studying the mechanisms of DBS in psychiatric disorders.

Although consistent with previous studies, our fMRI-NAc DBS model in the large animal has two major novel advancements, one being the custom MR radiofrequency (RF) receive coil, which has improved the signal-to-noise ratio (SNR) and thus played a significant role in allowing small signal changes to be measured (∼2.5X increase in SNR compared to regular transmit/receive birdcage coil; data not included). Second, we took a novel approach using a high-resolution 3-dimensional pig brain atlas [44], which allowed us to normalize the functional activation map and to apply general linear modeling in each subject group [37]. This method has the advantage of permitting regression analysis for every voxel across subjects, resulting in a high statistical power.

Importantly, our findings are in line with the anatomical connections of the NAc. NAc receives afferent projections from cingulate cortex, medial orbitofrontal cortex, and the granular insular cortex and sends efferent output to the dorsal substantia nigra, ventral tegmental area, and ventral pallidum [25]. The prelimbic areas of medial prefrontal cortex also project to the NAc [50]. These network connections may contribute to the explanation for the modulation of structures distal from the NAc/internal capsule region where the stimulation was applied.

### Stimulation Parameters and Optimization of Electrode Placement

In humans, the therapeutic effect of DBS depends on the stimulation parameters [46]. Likewise, in the pig model we observed the degree of activation to be voltage dependent, whereby increasing voltage from 1 V to higher voltages (3 V and 5 V) resulted in increased the volume of BOLD signal modulation in all animals. Likewise, variation in pulse width led to similar patterns of regions activated, but differences in the volume of BOLD signal modulation within some of these areas. Importantly, the voltage comparison and the pulse width comparison, which were performed in separate cohorts of animals, show a great deal of similarity in the pattern of regions activated, reflecting the repeatability of the results. Notably, however, there was a difference between the two cohorts in that the first group of animals (voltage comparison group; n = 7) demonstrated BOLD signal decrease in the thalamus whereas the second group of animals (pulse width comparison group; n = 3) demonstrated instead BOLD signal increase in the caudate nucleus, likely due to differences in the area of stimulation between the two groups.

Intriguingly, we observed both activation and deactivation of the thalamus and prefrontal with NAc/internal capsule DBS, dependent on both the individual pig and the stimulation parameters. This variation is likely due to differences in the targeting between experiments and highlights the importance of monitoring the functional sequelae of DBS to ensure proper electrode location. There is much debate surrounding the meaning of a decrease in BOLD signal. One hypothesis states that fMRI deactivation results from suppression of neuronal activity in the area of decreased signal [51]. However, the decrease in signal could alternatively be due to a vascular steal effect whereby areas of high activity divert blood flow away from the region in which the deactivation is observed [51] or due to a large increase in the metabolic needs of the tissue that exceeds the circulatory compensation [52]. As yet, we do not know whether a decrease in BOLD signal portends a different clinical outcome than an increase in BOLD signal but this will be an interesting avenue to investigate in follow-up clinical studies.

Finally, we investigated the issue of electrode placement with regard to the side in which the electrode is implanted. Although in human OCD patients there is some suggestion that the laterality of a neurosurgical intervention is a significant factor, with right-sided lesions being more effective than left-sided lesions [47], we observed similar activation patterns on either side, ipsilateral to the site of stimulation. This may be due to species differences between the human and the pig brain, with the human brain being more highly lateralized. Alternatively, it is possible that there are differences on a functional level that cannot be resolved by fMRI.

### Clinical Implications for Psychiatric Disorders

Importantly, this study demonstrates the effectiveness of NAc/internal capsule DBS in modulation of several highly clinically relevant brain regions. First, the insula is a fascinating brain area, which has been implicated in the processing of a wide variety of stimuli [53], [54]. The insula, buried in the lateral sulcus and covered by the operculum, is interconnected with both the prefrontal cortex and the cingulate gyrus [55], both of which showed concurrent activity in the present study. Furthermore, insula has a role in the pathophysiology of several psychiatric diseases. For example, hypoactivation in the insula is one fairly consistent alteration in activity present in major depressive disorder [56], and hyperactivation of the insula has been demonstrated during symptom provocation in OCD [57]. Therefore, the hypothesis that NAc/internal capsule DBS modulation of activity in insula contributes to the therapeutic effect of DBS for psychiatric disorders is intriguing and requires further study.

We also observed significant activation in prefrontal regions that was consistent across animals. This is consistent with what is known about the neuroanatomy of projections to the nucleus acumbens, namely that prelimbic areas of medial prefrontal cortex project to the NAc [50]. Additionally, these findings are clinically interesting, as Major Depressive Disorder is typically characterized by a decrease in activity in the prefrontal cortex [58] whereas OCD patients typically exhibit hyperactivity in prefrontal cortex at rest [59], and both these types of patients have been shown to benefit from NAc DBS. Furthermore, correction of prefrontal cortex activity to normal is correlated with symptom improvement in depression and OCD [60], [61]. Although the results in the present study represent for the most part an increase in activity in the prefrontal cortex, the results were obtained in a normal animal model and in an acute situation. It is conceivable that prefrontal cortex is differentially modulated in different pathological states, and that NAc/internal capsule stimulation potentially corrects this pathologic activity.

Likewise, the cingulate cortex is implicated in a range of disorders, many of which are responsive to NAc/internal capsule DBS. In Tourette’s syndrome, tic generation is associated with BOLD signal activation in the anterior cingulate [62]. Similarly, increases in cerebral regional blood flow have been detected with symptom provocation in OCD [63]. Finally, the level of metabolic activity in the anterior cingulate cortex can predict drug treatment response in major depressive disorder [64]. Therefore, modulation of any of these areas has the potential to significantly alter cognitive functioning; the combination of activation of multiple areas key to psychiatric illness makes the NAc an important target for investigation in the treatment of these disorders.

### Limitations and Future Directions

Although these results were obtained in a non-disease state animal model, we believe because of the similarity between the human and the pig brain among the deep brain structures as well as the similarity to the human surgical procedure, that these results are likely reflective of the effect of NAc/internal capsule DBS on the human circuitry. However, we acknowledge that although the deep structures of pig brain are closer in size to the human than is the rodent brain, there are still differences in the size of the NAc. Therefore, we utilized a human Medtronic 3389 stimulating electrode which has more closely spaced contacts (0.5 mm gap size) than the Medtronic 3387 (1.5 mm gap size) typically used in human DBS procedures in North America and stimulated only the 0,1 contacts [37]. Despite these precautions, it is possible that stimulation covered areas surrounding the NAc as well and we plan to use miniaturized DBS leads in the future [65].

Furthermore, our use of the pig animal model necessarily precluded imaging in the awake state. It is possible the awake state will have differential activation from the anesthetized state shown here. However, previous animal studies utilizing isoflurane anesthesia have shown significant BOLD signal responses to electrical stimulation and other stimuli [37], [66], [67], [68], [69].

Additionally, the present study was performed with acute stimulation delivered in a block paradigm. With this design, we were able to determine the BOLD response to variation in stimulation parameters and to trace stimulation-evoked neural network activation. However, it is possible that with chronic stimulation, such as is used in clinical situations, there will be differential modulation of activity.

### Conclusions

Overall, our results suggest that NAc/internal capsule DBS results in modulation of psychiatrically important brain areas notably the prefrontal cortex, cingulate and insula cortex, which may underlie the therapeutic effect of NAc DBS in psychiatric disorders. The results also suggest that the swine model for DBS fMRI, which conforms to human implanted DBS electrode configurations and human neuroanatomy, may be a useful platform for translational studies investigating the global neuromodulatory effects of DBS.
